# Transcriptome analysis around the onset of strawberry fruit ripening uncovers an important role of oxidative phosphorylation in ripening

**DOI:** 10.1038/srep41477

**Published:** 2017-02-14

**Authors:** Qing-Hua Wang, Cheng Zhao, Miao Zhang, Yu-Zhong Li, Yuan-Yue Shen, Jia-Xuan Guo

**Affiliations:** 1 Beijing Key Laboratory for Agricultural Application and New Technique, College of Plant Science and Technology, Beijing University of Agriculture, Beijing 102206, China; 2 Beijing Yuanquanyike Biological Technology Company, Beijing 100197, China; 3 Water Resources and Dryland Farming Laboratory, Institute of Agricultural Environment and Sustainable Development, Chinese Academy of Agricultural Sciences, Beijing 100081, P. R. China

## Abstract

Although much progress has been made towards understanding the ripening of non-climacteric fruit using the strawberry as a model plant, the defined molecular mechanisms remain unclear. Here, RNA-sequencing was performed using four cDNA libraries around the onset of ripening, and a total of 31,793 unigenes and 335 pathways were annotated including the top five pathways, which were involved in ribosome, spliceosome, protein processing, plant-pathogen interaction and plant hormone signaling, and the important DEGs related to ripening were annotated to be mainly involved in protein translation and processing, sugar metabolism, energy metabolism, phytohormones, antioxidation, pigment and softening, especially finding a decreased trend of oxidative phosphorylation during red-coloring. VIGS-mediated downregulation of the pyruvate dehydrogenase gene *PDHE1α*, a key gene for glycolysis-derived oxidative phosphorylation, could inhibit respiration and ATP biosynthesis, whilst promote the accumulation of sugar, ABA, ETH, and PA, ultimately accelerating the ripening. In conclusion, our results demonstrate that a set of metabolism transition occurred during green-to-white-to-red stages that are coupled with more-to-less DEGs, and the oxidative phosphorylation plays an important role in the regulation of ripening. On the basis of our results, we discuss an oxidative phosphorylation-based model underlying strawberry fruit ripening.

The ripening of fleshy fruit is a complex developmental process that involves many gene expression-controlled changes in texture, colour, flavour, and aroma. It is known that the process of climacteric fruit ripening is controlled by ethylene perception and signalling transduction^1^. Although much progress has now been made towards understanding the ripening process for non-climacteric fruit through abscisic acid (ABA) metabolism and signalling in strawberries^2^,^3^,^4^ (*Fragaria ananassa*), the defined molecular mechanisms remain unclear.

The strawberry is an ideal model plant for the study of non-climacteric fruit ripening^2^,^5^. In recent years, it is demonstrated that the ripening of strawberries is not only governed largely by ABA through a core signalling mechanism with the ABA-FaPYR1-FaPP2C-FaSnRK2 cascade^2^,^6^,^7^,^8^,^9^,^10^,^11^,^12^, but also involved in several other plant hormones including brassinosteroids (BR)^13^, jasmonate (JA)^14^, ethylene (ETH)^15^,^16^, and gibberelic acid (GA)^17^. Therefore, Shen and Rose (2015) consider that the regulation of non-climacteric ripening, to a large extent, is rather complex^4^.

Notably, transcriptional analyses of strawberries by expressed sequence tags (ESTs) and microarrays have found that ripening-related genes are involved in anthocyanin biosynthesis, cell wall degradation, sucrose and lipid metabolism, cellular transport, phosphatases, protein kinases, and transcription factors^18^,^19^,^20^. These findings have also been confirmed by proteomic analysis^11^,^21^. MicroRNA analysis has indicated that NAC transcription factors, auxin response factors (ARF), and Myb transcription factors are also involved in the regulation of strawberry fruit ripening and senescence^22^. More importantly, numerous groups of genes related to fruit development have been identified. One such group comprises genes involved in ripening, including FaSHP^10^ (SHATTERPROOF-like, C-type MADS-box gene), FaMADS9^23^ [A SEPALLATA(SEP)4-like gene), FaCDPK1^24^ (a calcium-dependent protein kinase), an aldolase gene^25^, FaCCD1^26^ (a carotenoid cleavage dioxygenase class 1 enzyme gene), FaGAST1 and FaGAST2^27^(cysteine-rich domain proteins), and FaCAD1^28^,^29^ (cinnamyl alcohol dehydrogenase). Another group consists of the anthocyanin biosynthesis-related genes, including *FaMYB10, FaMYB1, FaPA, FaCHS, FaCHI, FaF3H, FaDFR, FaANS, FaUFGT*, and *FaPRX27*^9^,^30^,^31^,^32^,^33^,^34^. A third group is the softening-related genes, which inlude *FaPG1*^35^,^36^, *FaPL* and *FaPE1*^37^,^38^, *FaEG1* and *FaEG3*^39^,^40^, *FaRGLyase1*^41^, *FaXyl1*^42^,^43^, *FaGAL1*^44^, *FaEXP1, FaEXP2* and *FaEXP5*^45^, and *FaPIP1* and *FaPIP2*^46^,^47^,^48^. Finally, several genes related to volatiles have been identified, including *FaAAT1* and *FaAAT2*^49^,^50^,^51^,^52^,^53^, *FaADH* and *FaPDC*^54^, *FaOM*T^55^,^56^,^57^, *FaNES1*^58^, *FaFAD1*^59^, *FaEGS*1^60^, and *FaGT2*^61^.

The regulation of strawberry fruit ripening involves many phytohormones, transcription factors, cis-elements, and metabolic enzymes. This suggests that the ripening-regulated molecular mechanisms of non-climacteric fruit, in contrast to those of climacteric fruit regulated by ethylene, is highly complex. Given that one obvious sign of the onset of strawberry fruit ripening is the development of red colouration, transcriptome-based RNA sequencing carried out at the reddening stage may provide insight into the molecular mechanisms of non-climacteric fruit ripening. We constructed and sequenced the cDNA libraries of receptacles at the large green, white, initial-red and partial-red stages using the Illumina HiSeq 2000 platform. Based on our results, we outline an oxidative phosphorylation-based model for the onset of strawberry fruit ripening.

## Results

### RNA sequencing analysis and de novo assembly

To maximize the transcriptional data obtained from ripening strawberries, we prepared four mixed cDNA libraries from ten uniformly sized fruits at each of four stages of ripeness for RNA sequencing using the Illumina HiSeq 2000 instrument^62^. Each sequenced sample yielded 100 bp reads from paired-end sequencing of cDNA fragments. After quality assessment and data clearance, 5.58–7.71 billion (G) reads with more than 90% Q20 bases (i.e., an average base quality greater than 20) were retained as high-quality reads for each library and used in subsequent analyses (Supplemental Table 1). The average ‘G + C’ content of the above was 48% [46%, 50%, 49%, and 48% for CM1 (large green), CM2 (white), CM3 (initial-red), and CM4 (partial-red) libraries, respectively] (Supplemental Table 1).

All valid reads were combined to perform de novo assembly by the paired-end method with Trinity software^63^ (trinityrnaseq_r20131110 version). A total of 98,848 unigenes were obtained, among which 24,358 genes were longer than 1 kb. An overview of the assembled transcripts and unigenes are presented in Supplemental Table 2. The length distributions of unigenes are shown in Supplemental Figure 1. These results demonstrate the effectiveness of Illumina pyrosequencing in rapidly capturing a large portion of the transcriptome.

### Sequence annotation

We used several complementary approaches to annotate the assembled sequences. The unigenes were compared against diverse protein databases, including the National Center for Biotechnology Information (NCBI) non-redundant protein (NR) database, SWISS-PROT, TrEMBL, Cdd, pfam, KOG database, Kyoto Encyclopedia of Genes and Genomes (KEGG), and Gene Ontology (GO). The resulting functional annotations are listed in Supplemental Table 3 and provide the best unigenes with an E-value of 1e^−5^ and identity of 30%. We performed a sequence similarity search against the NCBI NR and SWISS-PROT protein databases using the Basic Local Alignment Search Tool (BLAST) algorithm, specifying an E-value of less than 1e^−5^. This analysis indicated that of the 98,848 unigenes, 41,282 (41.76%) matched the NR database and 25,312 (25.61%) had similarity to proteins in the SWISS-PROT database. A total of 14,267 unigenes classified into 25 KOG annotations are provided in Supplemental Figure 2. The top three functional categories were signal transduction mechanisms, posttranslational modification/protein turnover/chaperones, and general function prediction only. Altogether, 43,769 (44.3%) unigenes were successfully annotated in NR, SWISS-PORT, TrEMBL, Cdd, pfam, KOG, KEGG, and GO. The high percentage (55.7%) of unmapped unigenes that could be assigned a putative function may be due mainly to the short sequence reads generated by the sequencing technology and the relatively short sequences of the resulting unigenes, most of which probably lack the conserved functional domains. Another possible explanation may be that some unigenes were non-coding RNAs or unknown.

Based on the BLAST UniProt annotation, we carried out GO analysis, which led to the annotation of 31,793 unigenes. The results comprise three classifications: “biological process,” “cellular component”, and “molecular function.” The most frequently associated GO terms for the biological process group were “metabolic process” (GO:) and “cellular process” (GO:); for the cellular component group, they were “cell” (GO:) and “cell part”; and for the molecular function group they were “binding” (GO:0005488) and “catalytic activity” (GO:0003824) (Supplemental Figure 3).

To analyze the KEGG pathway of unigenes, we used KAAS to obtain corresponding KO numbers. These KO numbers were then used to obtain corresponding KEGG pathways. By analysing the relationship between unigenes and enzymes in KEGG comment files and mapping them to the pathway, we created 335 pathway charts. This process predicted a total of 301 pathways represented by a total of 14,011 unigenes (Supplemental Table 4). The top five pathways were involved in ribosomes, spliceosomes, protein processing, plant-pathogen interaction, and plant hormone signal transduction (Supplemental Table 5).

### Gene expression patterns throughout fruit development

**Figure 1:**
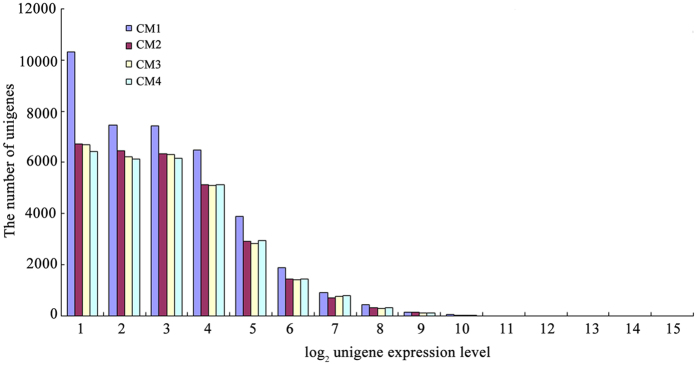
The distribution of gene expression levels. Total unigenes from each of four libraries were plotted as integrated log_2_ gene expression category.

We used the distribution of gene expression levels to evaluate the normality of the library data. Gene expression level was determined by calculating the number of reads and normalising to the RPKM. As shown in Fig. 1, the majority of mRNAs were expressed at low levels, whereas a small proportion was highly expressed. In CM1, most genes (10,307) have expression levels close to 1 (<1, namely log_2_ [2 RPKM]), while only 0.07% of the genes (27 of 39,086) were highly expressed at a level above 10. Compared with CM1, the distribution of the CM2, CM3, and CM4 libraries showed similar expression patterns; in other words, the later stages (white, initial-red, partial-red) were distinct from the large green stage, suggesting that the white stage might represent the onset of fruit ripening.

**Figure 2:**
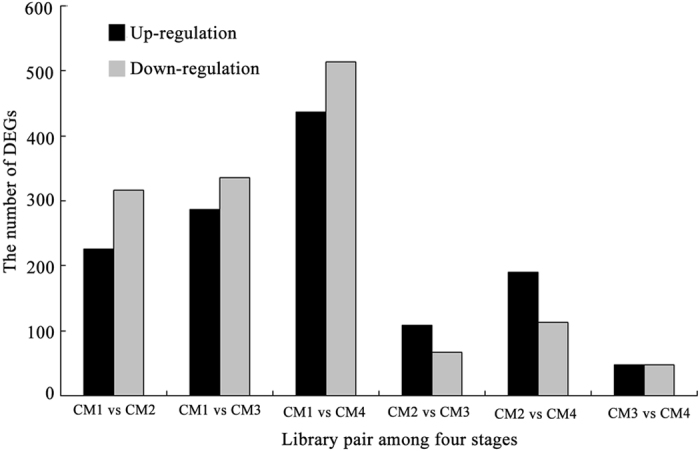
Differently expressed genes (DEGs) among the four-stage fruit. The number of up/down-regulated DEGs genes between library pairs was summarized by NCBI-NR, SWISS-PROT, TrEMBL, Cdd, pfam, KOG, KEGG and GO databases.

**Figure 3:**
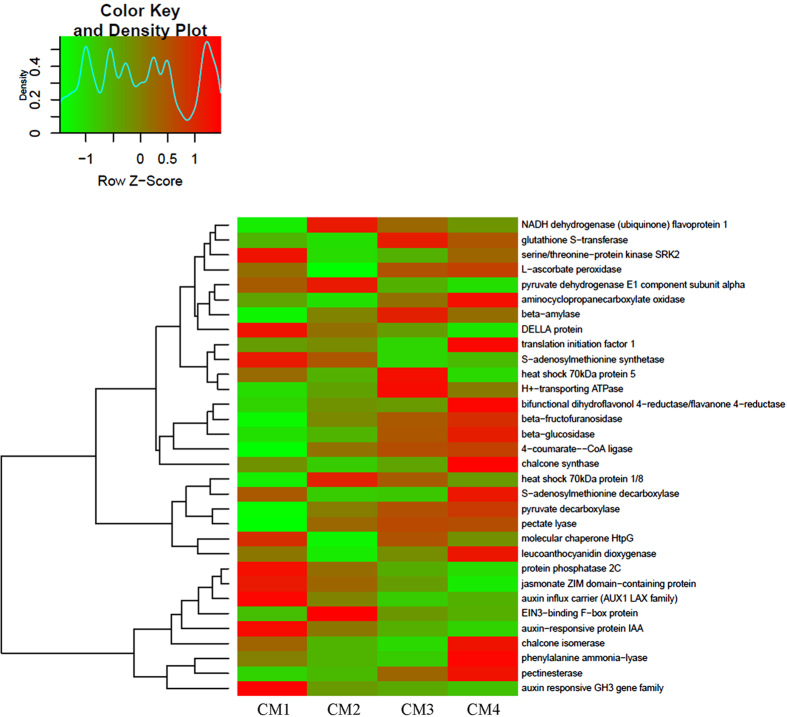
The Heatmap and cluster analysis of transcripts for the important genes related to the fruit ripening. Data for gene expression levels were normalized to z-score with the formula log10 (FPKM + 1) by color key and density plot, namely, the from-green-to-red color represents the value of gene expression from low to high during fruit ripening. The gene-coding proteins annotated in the relative pathways: **oxidative phosphorylation** [NADH dehydrogenase (ubiquinone) flavoprotein 1, H^+^-transporting ATPase]; **glutathione metabolism** [glutathione S-transferase (GST), L-ascorbate peroxidase (Vc)]; **abscisic acid** [ABA, serine/threonine-protein kinase SRK2, protein phosphatase 2C (PP2C)]; **protein processing** (heat shock 70 kDa protein 5, heat shock 70 kDa protein 1/8, molecular chaperone HtpG); **RNA transport** (translation initiation factor 1); **sugar metabolism** [pyruvate dehydrogenase E1 component alpha (PDH), beta-amylase, beta-fructofuranosidase, beta-glucosidase, pyruvate decarboxylase (PDC)]; **ethylene** [ETH, aminocyclopropanecarboxylate oxidase (ACO), EIN3-binding F-box protein (EBF), S-adenosylmethionine synthetase (SAMS)]; **Gibbérelline** (GA, DELLA protein); **flavonoid biosynthesis [**bifunctional dihydroflavonol 4-reductase/flavanone 4-reductase (DFR), chalcone synthase (CHS), chalcone isomerase (CHI), leucoanthocyanidin dioxygenase (LDOX), naringenin 3-dioxygenase (FHT)]; **phenylpropanoid biosynthesis** [4-coumarate-CoA ligase (4CL)], beta-glucosidase, phenylalanine ammonia-lyase (PAL)]; **pentose and glucuronate interconversions** [pectinesterase (PE), pectate lyase (PL)]; **jasmonate** [JA, jasmonate ZIM domain-containing protein (JAZ)]; **auxin** (IAA, auxin-responsive protein IAA, auxin influx carrier, auxin responsive GH3 gene family); **polyamines** [PA, S-adenosylmethionine decarboxylase (SAMDC)].

To analyze variation in gene expression throughout strawberry fruit development, the differentially expressed genes (DEGs) between each library pair (CM1-CM2, CM1-CM3, CM1-CM4, CM2-CM3, CM2-CM4, and CM3-CM4) were listed in Fig. 2.The results showed that more DEGs were detected when comparing CM2-CM4 to CM1, and among these DEGs, downrregulated genes were predominant, indicating that the onset of fruit ripening is coupled with many genes that were downregulated. Comparing CM3/CM4 with CM2, DEGs decreased sharply and upregulated genes were predominant, showing that these upregulated genes may therefore be involved in the onset of ripening. Notably, CM3 and CM4 had similar gene expression patterns. Some important DEGs related to ripening were listed in the Heatmap Fig. 3, mainly included oxidative phosphorylation (NADH, ATPase), plant hormones (ABA, IAA, GA, ETH, JA and PA), antioxidation (GST, Vc), protein translation and processing, sugar metabolism, pigment and softening. Taken together, these results suggest that a set of metabolism transition occurred during green-to-white-to-red stages, and the processes of fruit de-greening are more complex than those of fruit red-colouring, and that white fruit is a distinct stage.

### Enrichment pathway analysis of DEGs

**Figure 4:**
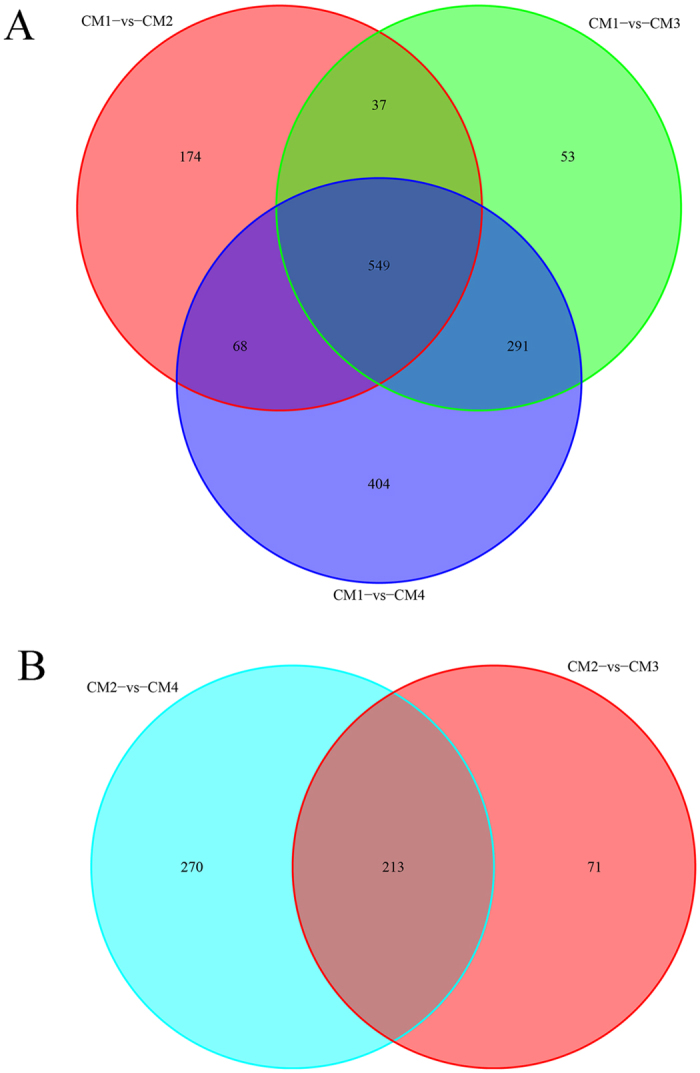
A Venn diagram for the different DEGs between each-stage-library pair. (**A**) Comparing CM2/CM3/CM4 to CM1; (**B**) Comparing CM3/CM4 to CM2.

We performed KEGG pathway enrichment analysis to categorise the biological functions of DEGs. We mapped all the genes to terms in the KEGG database. Comparing CM1 with CM2, CM3, and CM4, 41, 52, and 54 pathways were upregulated while 42, 48, and 54 pathways were downregulated, respectively. Comparing CM2 with CM3 and CM4, 36 and 39 pathways were upregulated while 42 and 43 pathways were downregulated, respectively. Comparing CM3 with CM4, 27 pathways were upregulated while 30 pathways were downregulated. Some DEGs specific to each stage are listed in Fig. 4, compared CM2/CM3/CM4 with CM1, 828, 930 and 1312 DEGs were found, respectively (Fig. 4A); compared CM3/CM4 with CM2, 284 and 483 DEGs were found, respectively (Fig. 4B); compared CM4 with CM3, only 192 DEGs were found.

The ten most DEG-rich pathways between library pairs are provided in Supplementary Table 6. Between CM1 and CM2, protein turnover and processing increased within pulp cells, as well as including oxidative phosphorylation, carbohydrate metabolism and the plant hormones (ABA, JA). The carbohydrate metabolism pathways included carbon fixation, glycolysis/gluconeogenesis, pentose and glucuronate interconversions, as well as starch degradation, sucrose accumulation, amino sugar, and nucleotide sugar metabolism related to fruit softening. Meanwhile, DNA turnover, photosynthesis, cell cycle, emergency reactions, and the plant hormones (IAA, GA, ETH and PA) signaling decreased within pulp cells.

Between CM1 and CM3/CM4, pulp cells maintained high levels of protein turnover and processing, oxidative phosphorylation, carbohydrate metabolism and the plant hormones (ABA, JA). Glutathione, phenylpropanoid/flavonoid, cysteine, and methionine metabolism were also active. Notably, cysteine and methionine metabolism were involved in ETH and PA biosynthesis. Carbon fixation, starch degradation, plant-pathogen reaction, and glyoxylate and dicarboxylate metabolism, and the plant hormones (including IAA and GA) signal transduction decreased.

Comparing CM2 to CM3/CM4, pulp cells sustained high levels of protein/DNA/RNA turnover and processing, as well as glutathione, flavonoid, sucrose metabolism and the plant hormones (ABA, JA, ETH and PA). Meanwhile, photosynthesis, carbon fixation, and the plant hormone (GA and IAA) signal transduction, as well as cysteine, methionine, and starch metabolism, decreased. Notably, oxidative phosphorylation metabolism also decreased.

Comparing CM3 to CM4, pulp cells sustained high levels of phenylpropanoid/flavonoid biosynthesis, carbohydrate metabolism (involved in pectate lyase, pectinesterase and beta-glucosidase), cysteine and methionine metabolism and the plant hormones (ABA, JA, ETH and PA). Porphyrin and chlorophyll metabolism, related to chlorophyll degradation, were also sustained at high levels. Photosynthesis, carbon fixation, and the plant hormone (GA and IAA) signal transduction decreased, as well as starch metabolism and oxidative phosphorylation.

Together, these results show that the processes of strawberry fruit ripening involve a decrease in oxidative phosphorylation and the plant hormone (GA and IAA) signaling, while an increase in carbohydrate, phenylpropanoid, flavonoid, glutathione, methionine and the plant hormone (ABA, JA, ethylene, and PA) signaling. The fact that the oxidative phosphorylation decreases at red-coloring, suggests a regulatory role of oxidative phosphorylation in ripening.

### Downregulation of PDHE1α by VIGS promotes strawberry fruit red-colouring

Given that the white stage represents the onset of fruit ripening and only several genes have an increased expression in this staged, including NADH dehydrogenase (ubiquinone) flavoprotein 1, pyruvate dehydrogenase E1 component alpha, heat shock 70 kDa protein 1/8 and EIN3-binding F-box protein (Fig. 3). It is known that pyruvate dehydrogenase contributes to linking the glycolysis metabolic pathway with the citric acid cycle and releasing energy via NADH dehydrogenase-mediated oxidative phosphorylation, and its E1 subunit is considered to be the rate-limiting step for the pyruvate dehydrogenase^64^.

**Figure 5:**
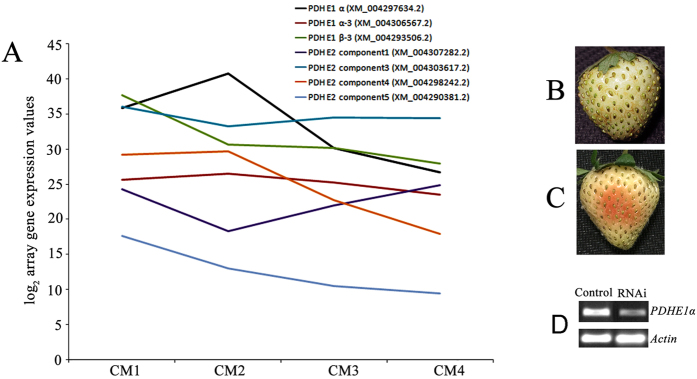
RNA-sequencing analysis of pyruvate dehydrogenase gene family and silencing of *PDHE1α* by VIGS in the fruit. (**A**) RNA-sequencing analysis of pyruvate dehydrogenase gene family. (**B,C**) Twenty large green fruit were used for treatment and control tests, respectively. One week after inoculation, phenotypes were observed for the control fruit (**B**) and RNAi fruit (**C**), respectively. (**D**) SqRT-PCR analysis of the transcripts of *PDHE1α* in receptacles of control fruit and RNAi fruit. *Actin* was used as a reference gene.

To further explore the role of oxidative phosphorylation in strawberry fruit development, we used the transcriptome data to investigate the mRNA expression levels of pyruvate dehydrogenase. The results showed that the E1 component subunit alpha of pyruvate dehydrogenase gene, *PDHE1α* (GenBank NO. XM_004297634.2), had an increased expression in white stage, and then followed a high-to-low pattern during strawberry fruit ripening (Fig. 5A). In order to investigate a role of the *PDHE1α* in the fruit ripening, the *PDHE1α* gene expression was silenced using syringe-infiltrated a mixture of *Agrobacterium* strain GV3101 cultures containing pTRV1 and pTRV2 carrying a 589-bp fragment of the gene (pTRV2-PDHE1α) into large green fruit in a 1:1 ratio. Control fruits were infiltrated only with TRVs alone. One week after infiltration, control fruit remained white (Fig. 5B); in contrast, RNAi fruit turned red (Fig. 3C), and *PDHE1α* transcripts were downregulated compared to the control (Fig. 5D). The results that downregulation of *PDHE1a* expression stimulated the fruit ripening, making PDHE1a a negative regulator of strawberry fruit ripening.

### Alteration of PDHE1α expression affects the ripening-related physiological parameters

To further elucidate the role of *PDHE1α* in the regulation of strawberry fruit ripening, we assayed ripening-related physiological parameters in both RNAi and control fruit, including plant hormones (ABA, GA, IAA, JA, ETH, and PA), soluble solids (sugar) concentration, biochemical measurements of oxidative phosphorylation (ATP), and respiration rate. The levels of ABA, ETH, PA, and soluble solids concentration were all remarkably high in RNAi fruit compared with control fruit (Table 1). In contrast, the levels of IAA, GA_3_, and JA showed non-significant changes between the RNAi and control fruit. Notably, both ATP content and respiration rate were markedly lower in RNAi fruit compared with control fruit. These results suggest that downregulation of *PDHE1α* expression inhibits respiration and ATP biosynthesis, resulting in an increase in soluble solids concentration and an accumulation of ABA, ETH, and PA, which ultimately promotes the ripening in RNAi fruit.

## Discussion

While the molecular mechanisms of strawberry fruit ripening are highly complex, the external signs (de-greening and red-colouring) are very simple. On the basis of our transcriptome-based RNA-sequencing analysis around the onset of ripening using large green, white, initial-red, and partial-red fruit, the previous and our data indicate that the pyruvate dehydrogenase E1α (PDHA1) is the first component enzyme of the pyruvate dehydrogenase (PDH) complex that catalyzes the overall conversion of pyruvate to acetyl-CoA, which is subsequently used in cellular respiration by both the citric acid cycle and oxidative phosphorylation to generate ATP, so this E1α subunit is considered to be the rate-limiting step for the pyruvate dehydrogenase complex^64^,^65^. The downregulation of PDHE1α expression inhibits respiration, ATP biosynthesis, and ABA and ETH accumulation (Fig. 5 and Table 1), to some extent, suggesting that a link between oxidative phosphorylation and strawberry fruit ripening is existed. Further explain is as follows:

It is known that oxidative phosphorylation play a central role in supplying the carbon skeleton and motive force for biochemical reactions, and that pyruvate dehydrogenase is a key step in the oxidative phosphorylation stage of sugar metabolism^66^. Our RNA-sequencing analysis indicated that the mRNA expression level of the E1 component subunit alpha of the pyruvate dehydrogenase gene *PDHE1α* follows a trend similar to that of oxidative phosphorylation activity at the onset of strawberry fruit ripening (Fig. 5). Interestingly, the downregulation of *PDHE1α* expression could inhibit respiration and ATP biosynthesis, whilst also promoting the accumulation of sugar, ABA, ETH, and PA, ultimately accelerating fruit ripening (Table 1). These results are consistent with previous reports on the roles of ABA, ETH, and sugar in the regulation of strawberry fruit ripening^2^,^7^,^8^,^9^,^10^,^11^,^12^,^67^,^68^.

On the one hand, glycolysis-derived oxidative phosphorylation provides the required carbon and energy fluxes for fruit development; on the other, cellular respiration through oxidative phosphorylation exhausts carbohydrates that provide the basis for fruit development. In the present study, the number of the differentially expressed genes (DEGs) is remarkable lower after white stage (comparing CM3/CM4 with CM2) in comparison to de-greening stages (comparing CM3/CM4/CM2 with CM1; Fig. 2, Supplemental Table 6), suggesting that the processes of early development are more complex (more DEGs) before white stages, once on the set of red-coloring in white fruit, the processes of ripening is relative simple (less DEGs). To a large extent, a metabolism transition occurred during green-to-white-to-red stages. In response to this change, fruit decreased oxidative phosphorylation intensity, which in turn promoted the accumulation of sugar, ABA, and ETH (Table 1), which can accelerate fruit ripening^7^,^67^.

**Figure 6:**
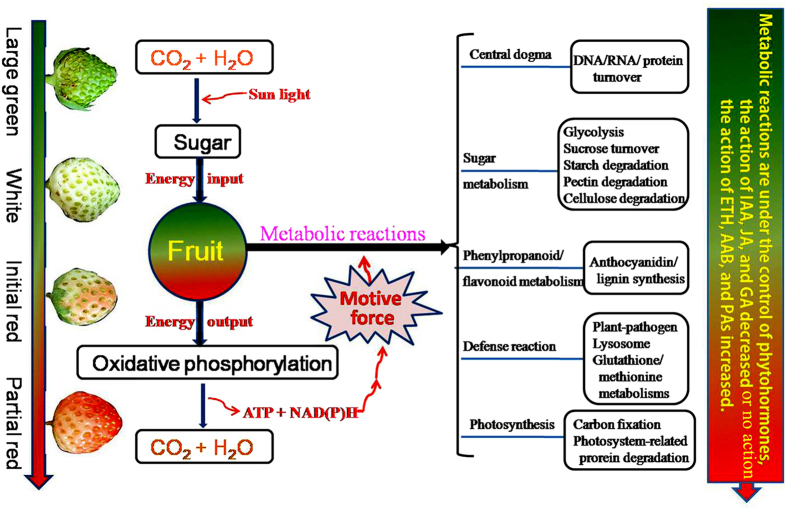
A model for the processes of strawberry fruit ripening at molecular level. With the action of sun energy, CO_2_ and H_2_O can be transformed into sugar by photosynthesis in chloroplast of leaves and fruit, the sugar can be turned into CO_2_ and H_2_O by oxidative phosphorylation to produce the motive force [ATP and NADH] for metabolic reactions. Coupled with the fruit de-greening and red-coloring, protein/DNA/RNA turnover always follows, the increased-sugar metabolisms are involved in glycolysis, sucrose turnover, and atarch, pectin and cellulose; the increased-phenylpropanoid/flavonoid metabolisms are mainly involved in anthocyanidin and lignin synthesis; The defense reactions are involved in plant-pathogen interaction, lysosome, glutathione, and methionine metabolisms. The cellular global metabolisms are under the programmed regulation of plant hormones, the action of IAA, JA, and GA decreased while the action of ethylene, ABA, and PA increased.

On the basis of the available data, we have tentatively outlined the processes of strawberry fruit ripening at the molecular level. With the action of sun energy, CO_2_ and H_2_O can be transformed into sugar by carbon fixation in the chloroplasts of leaves and fruit. Sugar can be turned into CO_2_ and H_2_O by oxidative phosphorylation in order to produce the motive force for biochemical metabolic reactions in fruit. During fruit development, protein/DNA/RNA turnover and oxidative phosphorylation follow a decreasing trend that corresponds to DEG variation (Fig. 2 and Table 1). It is interesting to note that, most upregulated DEGs were involved in sugar accumulation, fruit softening, and pigment biosynthesis (phenylpropanoid/flavonoid metabolism), while the capacity of fruit for carbon fixation and resistance decreased (Fig. 3 and Table 1). In response to these changes, fruit cells accelerate lysosome action and evoke cellular antioxidant action through glutathione and methionine metabolism^20^,^69^. Undoubtedly, cellular metabolism is regulated by plant hormones in response to fruit developmental cues: coupled with the onset of the fruit ripening, the action of ABA, ethylene, and polyamine increased (Fig. 6).

The present study provides the first description of an oxidative phosphorylation-based model for strawberry fruit ripening based on transcriptome data. Given the complexity of non-climacteric fruit ripening, the roles of aerobic respiration and anaerobic respiration in the regulation of fruit ripening are subjects for future study.

## Materials and Methods

### Plant material

Octoploid strawberry (*Fragaria* × *ananassa* cv. Hongyan) plants were grown in a greenhouse (17–26 °C, relative humidity 70–90%, 14/10-h light/dark regime) in springtime during 2013–2014. Sixty flowers on 20 strawberry plants were tagged during flowering. Four fruit stages (large green, white, initial-red, and partial-red) were numbered from CM1 to CM4 and collected at 18, 24, 27, and 29 days after anthesis, respectively. Ten uniformly sized fruits were sampled at every stage (one replicate). After removing the achenes (seeds), the receptacle (pulp) was cut into 0.5–0.8 cm^3^ cubes and quickly stored in liquid nitrogen.

### RNA extraction, library construction, and RNA-seq

Three fruits were randomly taken out from the frozen samples for every RNA isolation and cDNA synthesis. RNA was extracted using the RNeasy plant mini kit (Qiagen, Dusseldorf, Germany) from the receptacles of CM1-CM4 fruit. DNase digestion with the RNase-Free DNase set (Qiagen) was performed to remove contaminating DNA, and the RNA samples were then processed using the RNA library prep kit (New England BioLbs, Ipswich, MA, USA) (NEB) and sequenced with the Illumina HiSeq2000 platform. The experiments were repeated three times.

### Sequence data analysis and assembly

The raw reads were filtered with the FASTQ_Quality_Filter tool from the FASTX-toolkit. Reads with more than 35 bp having a quality score higher than 20 were kept^70^. All valid reads were combined to perform de novo splicing by the paired-end method with Trinity software^71^. The longest transcripts per locus were used as a unigene.

### Functional annotation

Several complementary approaches were utilised to annotate the unigenes. Searches were conducted using the Basic Local Alignment Search Tool (BLASTX)^72^. The unigenes were compared against the NCBI NR, SWISS-PROT, TrEMBL, Cdd, pfam, and KOG databases with an E-value of 1e-5 and identity of 30%^73^. Functional annotation by gene ontology terms (GO, http://www.geneontology.org) was carried out using the Blast2GO software^74^,^75^. To achieve KEGG orthology (KO) assignment^76^, the Kyoto Encyclopedia of Genes and Genomes (KEGG) pathways were assigned to sequences using the online KEGG Automatic Annotation Server (KAAS) (http://www.genome.jp/kegg/kaas/)^77^,^78^. The output of KEGG analysis includes KO assignments and KEGG pathways^77^,^78^.

The bowtie2–2.2.2 software was used to compare reads with unigenes using the single-end mapping method^71^,^79^. To compare the levels of unigene expression among the four libraries, the transcript level of each expressed unigene was calculated and normalised to the reads per kilobase of the exon model per million mapped reads^80^ (RPKM). The significance of differential unigene expression was determined by using the Chi-squared test with a threshold of P = 0.05. P-values were adjusted to account for multiple testing by using the false discovery rate (FDR) and assigned error ratio Q-values (<0.05)^81^. The unigenes with an adjusted P-value of <0.05 and an absolute value of log_2_ (expression fold change) >1 were deemed to be differentially expressed, while the unigenes with an FDR-adjusted P-value of <0.05 were considered statistically significant.

### VIGS and SqRT-PCR analysis

Following the method of Jia *et al*.^7^. (2011), a 589-bp cDNA fragment of *PDHE1α* was amplified using primers (sense 5′- TCTAGAACCACTGCACCTTCCTCG -3′, underlined is *Xba* І, and antisense 5′- CTCGAGTCACGCTCCTG TCTCACG -3′, underlined is *Xho* І) cloned into the virus vector *Xba* І-*Xho* І-cut pTRV2. *Agrobacterium* strain GV3101 containing the pTRV1, pTRV2, or pTRV2 derivatives pTRV2-PDHE1α_589_ was used for RNAi (Fig. 5). *Agrobacterium-*mediated TRV infiltration by syringe injection with a needle into strawberry fruits was performed as described by Jia *et al*.^7^. (2011). For semi-quantitative RT-PCR (SqRT-PCR) analysis of *PDHE1α* transcripts, the cDNA was used as a template for PCR amplification through 25 cycles and actin was used as a reference gene (sense 5′-CAGTTAGGAGAACTGGGTGC-3′ and antisense 5′-TGGGTTTGCTGGAGATGAT-3′). The experiments were repeated three times.

### Determination of fruit respiration, ethylene release rate, and soluble solid content

200 g of fruit were sealed in a 500 mL glass bottle at 25 °C. After 1 h, 10 mL of gas sample were used to measure respiration rate with an infrared carbon dioxide analyser (AMETEKMode lCD-3A) and ethylene release rate with a HP P5890a gas chromatograph. The soluble solids content of flesh was measured using a hand-held sugar measurement instrument (MASTER-100H, ATAGO Master, Japan), onto which fruit juice was applied to obtain a reading. Ten uniformly sized fruit were used for analysis. The experiments were repeated three times.

### ATP assay

ATP synthesis rates were measured using a luminometer (Glomax 96 Microplate Luminometer; Promega). 20 mL of isolated pulp chromoplasts were added to microplate wells containing 80 mL of luciferase/luciferin reagent (ENLITEN; Promega) and 80 mL of buffer (600 mM sorbitol, 10 mM TES, 2 mM MgCl2, 25 mM KH2PO4, and 0.33 mM EDTA, pH 7.4). Ten uniformly sized fruit were used for ATP synthesis measurement, and the experiments were repeated three times.

### Hormone analysis

In order to determine ABA, IAA, GA, and JA contents, 0.5 g receptacle samples were ground in a mortar and homogenised in extraction solution (80% methanol, v/v). Extracts were centrifuged at 10,000 × g for 20 min. The supernatant liquid was eluted through a Sep-Pak C18 cartridge (Waters, Milford, MA, USA) to remove polar compounds, and then stored at −20 °C for enzyme-linked immunosorbent assay (ELISA) according to the manufacturer’s instructions (China Agricultural University, Beijing, China). Ten uniformly sized fruit were used for detection, and the experiments were repeated three times.

### Polyamines assay

The polyamines assay was performed using high performance liquid chromatography (HPLC) with the Waters™ 996 photodiode Array Detector, Millennium 32 Chromatography software, and Diamond C18 columns (250 × 4.6 mm, 5 μm). The wavelength of detection was 230 nm, the flow rate was 1.2 mL/min, and and mobile phase was acetonitrile:methanol:water (3:4:3, v-v:v). 3 mL of precooled 5% perchloric acid (V/V) were added to 1 g samples, and the mixture was ground to homogeneity at 4 °C and centrifuged (15,000 rpm, 30 min, 4 °C) after 1 h extraction. 1 mL of supernatant, 7 μL of benzoyl chloride, and 1 mL 2 M NaOH solution were then added to 10 mL plastic centrifuge tubes. After 20 s of vortexing, the mixtures were incubated at 37 °C for 20 min in a water bath. 2 mL of saturated NaCl solution were added to the mixtures, which were then extracted with 2 mL diethyl ether after mixing. The mixture was centrifuged at 1,500 rpm for 5 min and 1 mL of ether phase was collected and dried under vaccum. The residuum was dissolved with 200 μL methanol and filtered through a 0.45 μm membrane, and 10 μL of filtrate were collected for injection. Ten uniformly sized fruit were used for analysis, and the experiments were repeated three times.

## Additional Information

**How to cite this article:** Wang, Q.-H. *et al*. Transcriptome analysis around the onset of strawberry fruit ripening uncovers an important role of oxidative phosphorylation in ripening. *Sci. Rep.* **7**, 41477; doi: 10.1038/srep41477 (2017).

**Publisher's note:** Springer Nature remains neutral with regard to jurisdictional claims in published maps and institutional affiliations.

## Supplementary Material

Supporting Information

## Figures and Tables

**Table 1:** Downregulation of *PDHE1α* expression affects the ripening-related physiological parameters.

Fruit	ABA (ng/g FW)	GA (ng/g FW)	IAA (ng/g FW)	JA (ng/g FW)	PAs (μg/g FW)	ETH (μL/kg h FW)	Solid soluble concentrations (g/100 g FW)	ATP (μg/g FW)	Respiration rate (mg/kg h)
RNAi	135.27 ± 9.20	11.15 ± 1.72	90.82 ± 7.31	24.17 ± 3.82	79.31 ± 5.51	0.28 ± 0.07	6.78 ± 1.03	17.05 ± 1.83	11.29 ± 1.54
	b	a	a	a	b	b	b	a	a
WT	109.34 ± 8.93	10.60 ± 1.13	87.95 ± 6.73	25.94 ± 2.59	55.45 ± 4.02	0.16 ± 0.04	4.53 ± 0.71	29.24 ± 2.58	19.06 ± 2.35
	a	a	a	a	a	a	a	b	b

## References

[b1] Alexander L. & Grierson D. Ethylene biosynthesis and action in tomato: a model for climacteric fruit ripening. J Exp Bot 53, 2039–2055 (2002). 12324528 10.1093/jxb/erf072

[b2] Li C. L., Jia H. F., Chai Y. M. & Shen Y. Y. Abscisic acid perception and signaling transduction in strawberry: A model for non-climacteric fruit ripening. Plant Signal &Behav 6, 1950–1953 (2011). 10.4161/psb.6.12.18024 PMC3337185 22095148

[b3] Cherian S., Figueroa C. R. & Nair H. 'Movers and shakers’ in the regulation of fruit ripening: a cross-dissection of climacteric versus non-climacteric fruit. J Exp Bot 65, 4705–4722 (2014). 24994760 10.1093/jxb/eru280

[b4] Shen Y. Y. & Rose J. K. C. ABA Metabolism and Signaling in Fleshy Fruits. *In*: Abscisic Acid: Metabolism, Transport and Signaling (Zhang D. P., ed.), pp. 271–286, Springer Science + Business Media Dordrecht (2015).

[b5] Given N. K., Venis M. A. & Grierson D. Hormonal regulation of ripening in the strawberry, a non-climacteric fruit. Planta 174, 402–406 (1988). 24221523 10.1007/BF00959527

[b6] Chai Y. M., Jia H. F., Li C. L., Dong Q. H. & Shen Y. Y. FaPYR1 is involved in strawberry fruit ripening. J Exp Bot 62, 5079–5089 (2011). 21778181 10.1093/jxb/err207

[b7] Jia H. F. . Abscisic acid plays an important role in the regulation of strawberry fruit ripening. Plant Physiol 157, 188–199 (2011). 21734113 10.1104/pp.111.177311 PMC3165869

[b8] Jia H. F. . Sucrose functions as a signal involved in the regulation of strawberry fruit development and ripening. New Phytol 198, 453–465 (2013). 23425297 10.1111/nph.12176

[b9] Jia H. F. . Type 2C protein phosphatase ABI1 is a negative regulator of strawberry fruit ripening. J Exp Bot 64, 1677–1687 (2013). 23404898 10.1093/jxb/ert028 PMC3617833

[b10] Daminato M., Guzzo F. & Casadoro G. A SHATTERPROOF-like gene controls ripening in non-climacteric strawberries, and auxin and abscisic acid antagonistically affect its expression. J Exp Bot 64, 3775–3786 (2013). 23888065 10.1093/jxb/ert214 PMC3745736

[b11] Li L. . Quantitative proteomic investigation employing stable isotope labeling by peptide dimethylation on proteins of strawberry fruit at different ripening stages. J Proteomics 94, 219–239 (2013). 24075981 10.1016/j.jprot.2013.09.004

[b12] Han Y. . FaSnRK2.6, an ortholog of Open Stomata 1, is a Negative Regulator of Strawberry Fruit Development and Ripening. Plant Physiol. doi: 10.1104/pp.114.251314 (2015). PMC4348756 25609556

[b13] Chai Y. M. . Brassinosteroid is involved in strawberry fruit ripening. Plant Growth Regulation 69, 63–69 (2013).

[b14] Concha C. M. Methyl jasmonate treatment induces changes in fruit ripening by modifying the expression of several ripening genes in Fragaria chiloensis fruit. Plant Physiol Biochem 70, 433–444 (2013). 23835361 10.1016/j.plaphy.2013.06.008

[b15] Trainotti L., Pavanello A. & Casadoro G. Different ethylene receptors show an increased expression during the ripening of strawberries: does such an increment imply a role for ethylene in the ripening of these non-climacteric fruits? J Exp Bot 56, 2037–2046 (2005). 15955790 10.1093/jxb/eri202

[b16] Merchante C. . Ethylene is involved in strawberry fruit ripening in an organ-specific manner. J Exp Bot 64, 4421–4439 (2013). 24098047 10.1093/jxb/ert257 PMC3808323

[b17] Csukasi F. . Gibberellin biosynthesis and signalling during development of the strawberry receptacle. New Phytol 191, 376–390 (2011). 21443649 10.1111/j.1469-8137.2011.03700.x

[b18] Manning K. Isolation of a set of ripening-related genes from strawberry: their identification and possible relationship to fruit quality traits. Planta 205, 622–631 (1998). 9684364 10.1007/s004250050365

[b19] Bombarely A. . Generation and analysis of ESTs from strawberry (Fragaria xananassa) fruits and evaluation of their utility in genetic and molecular studies. BMC Genomics 11, 503 (2010). 20849591 10.1186/1471-2164-11-503 PMC2996999

[b20] Aharoni A. & O’Connell A. P. Gene expression analysis of strawberry achene and receptacle maturation using DNA microarrays. J Exp Bot 53, 2073–2087 (2002). 12324531 10.1093/jxb/erf026

[b21] Bianco L. . Strawberry proteome characterization and its regulation during fruit ripening and in different genotypes. J Proteomics 72, 586–607 (2009). 19135558 10.1016/j.jprot.2008.11.019

[b22] Xu X. . High-throughput sequencing and degradome analysis identify miRNAs and their targets involved in fruit senescence of Fragaria ananassa. PLoS One 8, e70959 (2013). 23990918 10.1371/journal.pone.0070959 PMC3747199

[b23] Seymour G. B. . A SEPALLATA gene is involved in the development and ripening of strawberry (Fragaria x ananassa Duch.) fruit, a non-climacteric tissue. J Exp Bot 62, 1179–1188 (2011). 21115665 10.1093/jxb/erq360 PMC3022409

[b24] Llop-Tous I., Domínguez-Puigjaner E. & Vendrell M. Characterization of a strawberry cDNA clone homologous to calcium-dependent protein kinases that is expressed during fruit ripening and affected by low temperature. J Exp Bot 53, 2283–2285 (2002). 12379799 10.1093/jxb/erf103

[b25] Schwab W., Aharoni A., Raab T., Pérez A. G. & Sanz C. Cytosolic aldolase is a ripening related enzyme in strawberry fruits (Fragaria x ananassa). Phytochemistry 56, 407–415 (2001). 11261572 10.1016/s0031-9422(00)00405-2

[b26] García-Limones C. . Functional characterization of FaCCD1: a carotenoid cleavage dioxygenase from strawberry involved in lutein degradation during fruit ripening. J Agric Food Chem 56, 9277–9285 (2008). 18778069 10.1021/jf801096t

[b27] Moyano-Cañete E. . FaGAST2, a strawberry ripening-related gene, acts together with FaGAST1 to determine cell size of the fruit receptacle. Plant Cell Physiol 54, 218–236 (2013). 23231876 10.1093/pcp/pcs167

[b28] Aharoni A. . Novel insight into vascular, stress, and auxin-dependent and -independent gene expression programs in strawberry, a non-climacteric fruit. Plant Physiol 129, 1019–1031 (2002). 12114557 10.1104/pp.003558 PMC166497

[b29] Blanco-Portales R. . Cloning, expression and immunolocalization pattern of a cinnamyl alcohol dehydrogenase gene from strawberry (Fragaria x ananassa cv. Chandler). J Exp Bot 53, 1723–1734 (2002). 12147722 10.1093/jxb/erf029

[b30] Aharoni A. . The strawberry FaMYB1 transcription factor suppresses anthocyanin and flavonol accumulation in transgenic tobacco. Plant J 28, 319–332 (2001). 11722774 10.1046/j.1365-313x.2001.01154.x

[b31] Salvatierra A., Pimentel P., Moya-León M. A. & Herrera R. Increased accumulation of anthocyanins in Fragaria chiloensis fruits by transient suppression of FcMYB1 gene. Phytochemistry 90, 25–36 (2013). 23522932 10.1016/j.phytochem.2013.02.016

[b32] Ring L. . Metabolic interaction between anthocyanin and lignin biosynthesis is associated with peroxidase FaPRX27 in strawberry fruit. Plant Physiol 163, 43–60 (2013). 23835409 10.1104/pp.113.222778 PMC3762661

[b33] Schaart J. G. . Identification and characterization of MYB-bHLH-WD40 regulatory complexes controlling proanthocyanidin biosynthesis in strawberry (Fragaria × ananassa) fruits. New Phyto 197, 454–467 (2013). 10.1111/nph.12017 23157553

[b34] Medina-Puche L. . MYB10 plays a major role in the regulation of flavonoid/phenylpropanoid metabolism during ripening of Fragaria x ananassa fruits. J Exp Bot 65, 401–417 (2014). 24277278 10.1093/jxb/ert377

[b35] Posé S. . Insights into the effects of polygalacturonase FaPG1 gene silencing on pectin matrix disassembly, enhanced tissue integrity, and firmness in ripe strawberry fruits. J Exp Bot 64, 3803–3815 (2013). 23873994 10.1093/jxb/ert210 PMC3745733

[b36] Paniagua C. . Fruit softening and pectin disassembly: an overview of nanostructural pectin modifications assessed by atomic force microscopy. Ann Bot 114, 1375–1383 (2014). 25063934 10.1093/aob/mcu149 PMC4195560

[b37] Castillejo C. . Pectin esterase gene family in strawberry fruit: study of FaPE1, a ripening-specific isoform. J Exp Bot 55, 909–918 (2004). 15020638 10.1093/jxb/erh102

[b38] Santiago-Doménech N. . Antisense inhibition of a pectate lyase gene supports a role for pectin depolymerization in strawberry fruit softening. J Exp Bot. 59, 2769–2779 (2008). 18522930 10.1093/jxb/ern142 PMC2486476

[b39] Trainotti L., Spolaore S., Pavanello A., Baldan B. & Casadoro G. A novel E-type endo-beta-1,4-glucanase with a putative cellulose-binding domain is highly expressed in ripening strawberry fruits. Plant Mol Biol 40, 323–332 (1999). 10412910 10.1023/a:1006299821980

[b40] Woolley L. C., James D. J. & Manning K. Purification and properties of an endo-beta-1,4-glucanase from strawberry and down-regulation of the corresponding gene, cel1. Planta 214, 11–21 (2001). 11762160 10.1007/s004250100577

[b41] Molina-Hidalgo F. J. . The strawberry (Fragaria x ananassa) fruit-specific rhamnogalacturonate lyase 1 (FaRGLyase1) gene encodes an enzyme involved in the degradation of cell-wall middle lamellae. J Exp Bot 64, 1471–1483 (2013). 23564958 10.1093/jxb/ers386

[b42] Martínez G. A., Chaves A. R. & Civello P. M. β-xylosidase activity and expression of a beta-xylosidase gene during strawberry fruit ripening. Plant Physiol Biochem 42, 89–96 (2004). 15283123 10.1016/j.plaphy.2003.12.001

[b43] Bustamante C. A., Rosli H. G., Añón M. C., Civello P. M. & Martínez G. A. β-Xylosidase in strawberry fruit: Isolation of a full-length gene and analysis of its expression and enzymatic activity in cultivars with contrasting firmness. Plant Sci 171, 497–504 (2006). 25193647 10.1016/j.plantsci.2006.05.011

[b44] Trainotti L., Spinello R., Piovan A., Spolaore S. & Casadoro G. β-Galactosidases with a lectin-like domain are expressed in strawberry. J Exp Bot 52, 1635–1645 (2001). 11479328

[b45] Dotto M. C., Martínez G. A. & Civello P. M. Expression of expansin genes in strawberry varieties with contrasting fruit firmness. Plant Physiol Biochem 44, 301–307 (2006). 16889972 10.1016/j.plaphy.2006.06.008

[b46] Mut P. . A fruit-specific plasma membrane aquaporin subtype PIP1 is regulated during strawberry (Fragaria x ananassa) fruit ripening. Physiol Plant 132, 538–551 (2008). 18248507 10.1111/j.1399-3054.2007.01046.x

[b47] Alleva K. . Cloning, functional characterization, and co-expression studies of a novel aquaporin (FaPIP2) of strawberry fruit. J Exp Bot 61, 3935–3945 (2010). 20663858 10.1093/jxb/erq210 PMC2935871

[b48] Yaneff A. . Heteromerization of PIP aquaporins affects their intrinsic permeability. Proc Natl Acad Sci USA 111, 231–236 (2014). 24367080 10.1073/pnas.1316537111 PMC3890845

[b49] Aharoni A. . Identification of the SAAT gene involved in strawberry flavor biogenesis by use of DNA microarrays. Plant Cell 12, 647–662 (2000). 10810141 10.1105/tpc.12.5.647 PMC139918

[b50] Yahyaoui F. E. . Molecular and biochemical characteristics of a gene encoding an alcohol acyl-transferase involved in the generation of aroma volatile esters during melon ripening. Eur J Biochem 269, 2359–2366 (2002). 11985619 10.1046/j.1432-1033.2002.02892.x

[b51] Beekwilder J. . Functional characterization of enzymes forming volatile esters from strawberry and banana. Plant Physiol 135, 1865–1878 (2004). 15326278 10.1104/pp.104.042580 PMC520758

[b52] González M. . Aroma development during ripening of Fragaria chiloensis fruit and participation of an alcohol acyltransferase (FcAAT1) gene. J Agric Food Chem 57, 9123–9132 (2009). 19731914 10.1021/jf901693j

[b53] Cumplido-Laso G. . The fruit ripening-related gene FaAAT2 encodes an acyl transferase involved in strawberry aroma biogenesis. J Exp Bot 63, 4275–4290 (2012). 22563120 10.1093/jxb/ers120

[b54] Ponce-Valadez M. & Watkins C. B. Fermentation and malate metabolism in response to elevated CO_2_ concentrations in two strawberry cultivars. Physiol Plant 134, 121–133 (2008). 18494736 10.1111/j.1399-3054.2008.01108.x

[b55] Wein M. . Isolation, cloning and expression of a multifunctional O-methyltransferase capable of forming 2,5-dimethyl-4-methoxy-3(2H)-furanone, one of the key aroma compounds in strawberry fruits. Plant J 31, 755–765 (2002). 12220266 10.1046/j.1365-313x.2002.01396.x

[b56] Lunkenbein S. . Up- and down-regulation of Fragaria x ananassa O-methyltransferase: impacts on furanone and phenylpropanoid metabolism. J Exp Bot 57, 2445–2453 (2006). 16798852 10.1093/jxb/erl008

[b57] Zorrilla-Fontanesi Y. . Genetic analysis of strawberry fruit aroma and identification of O-methyltransferase FaOMT as the locus controlling natural variation in mesifurane content. Plant Physiol 159, 851–870 (2012). 22474217 10.1104/pp.111.188318 PMC3375946

[b58] Aharoni A. . Gain and loss of fruit flavor compounds produced by wild and cultivated strawberry species. Plant Cell 16, 3110–3131 (2004). 15522848 10.1105/tpc.104.023895 PMC527202

[b59] Chambers A. H. . Identification of a strawberry flavor gene candidate using an integrated genetic-genomic-analytical chemistry approach. BMC Genomics 15, 217 (2014). 24742080 10.1186/1471-2164-15-217 PMC4023330

[b60] Aragüez I. . Eugenol production in achenes and receptacles of strawberry fruits is catalyzed by synthases exhibiting distinct kinetics. Plant Physiol 163, 946–958 (2013). 23983228 10.1104/pp.113.224352 PMC3793070

[b61] Lunkenbein S. . Cinnamate metabolism in ripening fruit. Characterization of a UDP-glucose:cinnamate glucosyltransferase from strawberry. Plant Physiol 140, 1047–1058 (2006). 16443693 10.1104/pp.105.074955 PMC1400576

[b62] Xiao-Jiao H., Yang-Dong W., Yi-Cun C., Li-Yuan L. & Qing-Ke W. Transcriptome sequencing and expression analysis of terpenoid biosynthesis genes in Litsea cubeba. Plos one 8, 1–14 (2013). 10.1371/journal.pone.0076890 PMC3793921 24130803

[b63] Grabherr M. G. . Trinity: reconstructing a full-length transcriptome without a genome from RNA-Seq data. Nat Biotechnol 29, 644–652 (2011). 21572440 10.1038/nbt.1883 PMC3571712

[b64] Sheng X. & Liu Y. Theoretical study of the catalytic mechanism of E1 subunit of pyruvate dehydrogenase multienzyme complex from Bacillus stearothermophilus. Biochemistry 52, 8079–8093 (2013). 24171427 10.1021/bi400577f

[b65] Ozden O. . SIRT3 deacetylates and increases pyruvate dehydrogenase activity in cancer cells. Free Radic Biol Med 76, 163–172 (2014). 25152236 10.1016/j.freeradbiomed.2014.08.001 PMC4364304

[b66] Mayers R. M., Leighton B. & Kilgour E. PDH kinase inhibitors: a novel therapy for Type II diabetes? Biochem Soc Trans 33, 367–370 (2005). 15787608 10.1042/BST0330367

[b67] Sun J. H. . New evidence for the role of ethylene in strawberry fruit ripening. Journal of Plant Growth Regulation 32, 461–470 (2012).

[b68] Merchante C. . Ethylene is involved in strawberry fruit ripening in an organ-specific manner. J Exp Bot 64, 4421–4439 (2013). 24098047 10.1093/jxb/ert257 PMC3808323

[b69] Wang Y., Xu F., Feng X. & MacArthur R. L. Modulation of Actinidia arguta fruit ripening by three ethylene biosynthesis inhibitors. Food Chem 173, 405–4013 (2015). 25466039 10.1016/j.foodchem.2014.10.044

[b70] Mingying L. . Transcriptome sequencing and de novo analysis for bamboo using the illumina platform. Plos one 7, 1–11 (2012). 10.1371/journal.pone.0046766 PMC3463524 23056442

[b71] Wang Z. . De novo assembly and characterization of root transcriptome using Illumina paired-end sequencing and development of cSSR markers in sweetpotato (Ipomoea batatas). BMC Genomics 11, 726 (2010). 21182800 10.1186/1471-2164-11-726 PMC3016421

[b72] Kent W. J. BLAT—The BLAST-Like Alignment Tool. Genome Res 12, 656–664 (2002). 11932250 10.1101/gr.229202 PMC187518

[b73] Altschul S. F. . Gapped BLAST and PSI-BLAST: a new generation of protein database search programs. Nucleic Acids Res 25, 3389–3402 (1997). 9254694 10.1093/nar/25.17.3389 PMC146917

[b74] Conesa A., Götz S., García-Gómez J. M., Terol J. & Talón M. Blast2GO: a universal tool for annotation, visualization and analysis in functional genomics research. Bioinformatics 21, 3674–3676 (2005). 16081474 10.1093/bioinformatics/bti610

[b75] Conesa A. & Götz S. Blast2GO: A comprehensive suite for functional analysis in plant genomics. Int J Plants Genom. doi: 10.1155/2008/619832 (2008). PMC2375974 18483572

[b76] Moriya Y. . KAAS: an automatic genome annotation and pathway reconstruction server. Nucleic Acids Res 35, 182–185 (2007). 10.1093/nar/gkm321 PMC1933193 17526522

[b77] Karp P. D., Paley S. & Romero P. The pathway tools software. Bioinformatics 18, 225–232 (2002). 10.1093/bioinformatics/18.suppl_1.s225 12169551

[b78] Kanehisa M., Goto S., Kawashima S., Okuno Y. & Hattori M. The KEGG resource for deciphering the genome. Nucleic Acids Res 32, 277–280 (2004). 10.1093/nar/gkh063 PMC308797 14681412

[b79] Ben L. & Salzberg S. L. Fast gapped-read alignment with Bowtie 2. Nature mathods 9, 357–359 (2012). 10.1038/nmeth.1923 PMC3322381 22388286

[b80] Mortazavi A., Williams B. A., McCue K., Schaeffer L. & Wold B. Mapping and quantifying mammalian transcriptomes by RNA-Seq. Nat Meth 5, 621–628 (2008). 10.1038/nmeth.1226 PMC13303166 18516045

[b81] Benjamini Yoav Y. D. The control of the false discovery rate in multiple testing under dependency. The annals of statistics 29, 1165–1188 (2001).

