# Supplementary material for: Transcriptome analysis around the onset of strawberry fruit ripening uncovers an important role of oxidative phosphorylation in ripening
Source: Sci Rep. 2017 Feb 14;7:41477. doi: 10.1038/srep41477 (PMC5307319; doi:10.1038/srep41477)
Supplement: Supporting Information [file srep41477-s1.doc]

**Supplemental Data**

**Title:** Transcriptome analysis around the onset of strawberry fruit ripening uncovers an important role of oxidative phosphorylation in ripening

**Subtitle:** Transcriptome analysis around the onset of strawberry fruit ripening

**Competing financial interests:** The authors declare no competing financial interests.

**Additional Information**

**Authors:** Qing-Hua Wang^1^*, Cheng Zhao^1^*, Miao Zhang^2^ *, Yu-Zhong Li^3^, Yuan-Yue Shen^1^**^ψ^**, Jia-Xuan Guo^1^**^ψ^**

**Address:** ^1^ Beijing Key Laboratory for Agricultural Application and New Technique, College of Plant Science and Technology, Beijing University of Agriculture, Beijing 102206, China

^2^ Beijing Yuanquanyike Biological Technology Company, Beijing 100197, China

^3^ Water Resources and Dryland Farming Laboratory, Institute of Agricultural Environment and Sustainable Development, Chinese Academy of Agricultural Sciences, Beijing 100081, P. R. China

* These authors contributed equally to this work.

**^ψ^**Author for correspondence, e-mail: [guojiaxuangjx@163.com](mailto:guojiaxuangjx@163.com); sfmn@tom.com

**The number of figures:** 6

**The number of tables:** 1

**The total word count and numbers:** 7348

Supplementary Table 1: Description of four *Fragaria ananassa* RNA-Seq libraries.

Supplementary Table 2: Summary of Illumina transcriptome assembly for strawberry

Supplementary Table 3: The statistic results of sequence mapping to the public database

Supplementary Table 4: The results of KEGG annotation

Supplementary Table 5: The top five pathways annotated with KEGG pathway analysis

Supplementary Table 6: List of ten more enriched pathways for up-regulated and down-regulated DEGs between library pairs

Supplementary Figure 1: Sequence assembly by all-unigene size distribution of strawberry unigenes.

Supplementary Figure 2: KOG function classification of consensus sequence.

Supplementary Figure 3: The result of comparison of GO classification.

**Supplementary Table 1 Description of four *F*. vesca RNA-Seq libraries**

| **Library name** | **Total reads** | **Total bases** | **G+C content (%)** | **Cycle Q20 (%)** |
| --- | --- | --- | --- | --- |
| **CM1** | 60,384,252 | 6,098,809,452 | 46 | 92.34 |
| **CM2** | 86,321,662 | 8,718,487,862 | 50 | 90.97 |
| **CM3** | 59,731,790 | 6,032,910,790 | 49 | 94.96 |
| **CM4** | 61,782,324 | 6,240,014,724 | 48 | 95.53 |

CM1, large green fruit; CM2, white fruit; CM3, initial red fruit; CM4, Partial red fruit.

**Supplementary Table 2 Summary of Illumina transcriptome assembly for strawberry**

| **Library name** | **Total reads** | **N50^a^** | **N90^b^** | **Total Length** | **Max Length** | **Min Length** | **Average Length** |
| --- | --- | --- | --- | --- | --- | --- | --- |
| **Transcript** | 239204 | 1590 | 424 | 239,905,623 | 15874 | 201 | 1002.93 |
| **Unigene** | 98848 | 1489 | 284 | 77,415,040 | 15874 | 201 | 783.17 |

^a^ N50, sorted the transcripts from long to short, then accumulated bases of transcripts in turn, when the total bases number reached half of total number of bases, the length of transcript, as well as unigenes. ^b^ N90 was counted in a similar way.

**Supplementary Table 3 The statistic results of sequence mapping to the public database**

| **Num** | **NR** | **SWISS-**  **PROT** | **TREMBL** | **CDD** | **PFAM** | **KOG** |
| --- | --- | --- | --- | --- | --- | --- |
| 98848 | 41282 | 25312 | 42107 | 23343 | 38306 | 14267 |
| 100% | 41.76% | 25.61% | 42.60% | 23.62% | 38.75% | 14.43% |

**Supplementary Table 4 The results of KEGG annotation**

| **Number of unigenes annotated** | **Number of enzymes annotated** | **Number of pathways mapped** |
| --- | --- | --- |
| 14011 | 997 | 301 |

**Supplementary Table 5 The top five pathways annotated with KEGG pathway analysis**

| **Pathway** | **Unigene number** |
| --- | --- |
| **ko03010, Ribosome** | 625 |
| **ko03040, Spliceosome** | 484 |
| **ko04141, Protein processing in endoplasmic reticulum** | 427 |
| **ko04626, Plant-pathogen interaction** | 410 |
| **ko04075, Plant hormone signal transdution** | 342 |

**Supplementary able 6**  List of first five pathways for up-regulated and down-regulated DEGs between library pairs

| **Pathway term** | **Pathway ID** | **DEGs test** | **P value** |
| --- | --- | --- | --- |
| **CM1 vs. CM2** | | | |
| **Pathways for up-regulated DEGs** | | | |
| Ribosome | ko03010 | 45 | 4.92E-05 |
| Oxidative phosphorylation | ko00190 | 30 | 6.87E-06 |
| Protein processing in endoplasmic reticulum | ko04141 | 19 | 0.257382973 |
| Carbon fixation in photosynthetic organisms | ko00710 | 19 | 1.92E-05 |
| Starch and sucrose metabolism | ko00500 | 17 | 0.091298615 |
| Glycolysis / Gluconeogenesis | ko00010 | 17 | 0.003464649 |
| Proteasome | ko03050 | 15 | 2.06E-07 |
| Amino sugar and nucleotide sugar metabolism | ko00520 | 13 | 0.011901347 |
| Lysosome | ko04142 | 13 | 0.000581877 |
| Pentose and glucuronate interconversions | ko00040 | 13 | 4.12E-05 |
| **Pathways for down-regulated DEGs** | | | |
| Protein processing in endoplasmic reticulum | ko04141 | 60 | 8.17E-17 |
| Photosynthesis | ko00195 | 44 | 2.21E-37 |
| Ribosome | ko03010 | 26 | 0.494112209 |
| Photosynthesis - antenna proteins | ko00196 | 20 | 2.66E-16 |
| Plant-pathogen interaction | ko04626 | 20 | 0.219398363 |
| Cell cycle | ko04110 | 20 | 0.002533181 |
| DNA replication | ko03030 | 19 | 3.12E-10 |
| Antigen processing and presentation | ko04612 | 19 | 3.17E-07 |
| Pyrimidine metabolism | ko00240 | 16 | 0.005577683 |
| Plant hormone signal transduction | ko04075 | 14 | 0.488221579 |
| **CM1 vs. CM3** | | | |
| **Pathways for up-regulated DEGs** | | | |
| Protein processing in endoplasmic reticulum | ko04141 | 42 | 2.87E-09 |
| Oxidative phosphorylation | ko00190 | 27 | 3.14E-05 |
| Starch and sucrose metabolism | ko00500 | 21 | 0.004176327 |
| Glycolysis / Gluconeogenesis | ko00010 | 21 | 2.70E-05 |
| Carbon fixation in photosynthetic organisms | ko00710 | 16 | 0.000237364 |
| Glutathione metabolism | ko00480 | 14 | 0.000289284 |
| Pentose and glucuronate interconversions | ko00040 | 13 | 1.81E-05 |
| Phagosome | ko04145 | 12 | 0.012085043 |
| Lysosome | ko04142 | 12 | 0.000912216 |
| Pyruvate metabolism | ko00620 | 12 | 0.006483415 |
| **Pathways for down-regulated DEGs** | | | |
| Photosynthesis | ko00195 | 41 | 2.07E-38 |
| Protein processing in endoplasmic reticulum | ko04141 | 21 | 0.020651994 |
| Photosynthesis - antenna proteins | ko00196 | 20 | 9.10E-19 |
| Plant hormone signal transduction | ko04075 | 20 | 0.003659762 |
| Ribosome | ko03010 | 17 | 0.714692855 |
| Glyoxylate and dicarboxylate metabolism | ko00630 | 14 | 5.77E-06 |
| Ubiquitin mediated proteolysis | ko04120 | 14 | 0.008540865 |
| Cell cycle | ko04110 | 13 | 0.032629239 |
| Starch and sucrose metabolism | ko00500 | 12 | 0.233993238 |
| Carbon fixation in photosynthetic organisms | ko00710 | 11 | 0.010179951 |
| **CM1 vs. CM4** | | | |
| **Pathways for up-regulated DEGs** | | | |
| Protein processing in endoplasmic reticulum | ko04141 | 33 | 0.000631217 |
| Starch and sucrose metabolism | ko00500 | 22 | 0.014862533 |
| Oxidative phosphorylation | ko00190 | 20 | 0.050256939 |
| Phenylpropanoid biosynthesis | ko00940 | 19 | 0.001232148 |
| Cysteine and methionine metabolism | ko00270 | 18 | 1.99E-05 |
| Glycolysis / Gluconeogenesis | ko00010 | 18 | 0.004316539 |
| Proteasome | ko03050 | 17 | 2.31E-08 |
| Lysosome | ko04142 | 17 | 1.23E-05 |
| Glutathione metabolism | ko00480 | 17 | 6.15E-05 |
| Flavonoid biosynthesis | ko00941 | 17 | 6.41E-10 |
| **Pathways for down-regulated DEGs** | | | |
| Photosynthesis | ko00195 | 48 | 8.09E-40 |
| Protein processing in endoplasmic reticulum | ko04141 | 46 | 2.65E-07 |
| Plant hormone signal transduction | ko04075 | 24 | 0.033880821 |
| Starch and sucrose metabolism | ko00500 | 23 | 0.029594429 |
| Plant-pathogen interaction | ko04626 | 23 | 0.225202906 |
| Photosynthesis - antenna proteins | ko00196 | 22 | 2.24E-17 |
| Glyoxylate and dicarboxylate metabolism | ko00630 | 21 | 6.86E-08 |
| Glycolysis / Gluconeogenesis | ko00010 | 21 | 0.001570279 |
| Ribosome | ko03010 | 21 | 0.966325864 |
| Carbon fixation in photosynthetic organisms | ko00710 | 19 | 0.000369877 |
| **CM2 vs. CM3** | | | |
| **Pathways for up-regulated DEGs** | | | |
| Protein processing in endoplasmic reticulum | ko04141 | 58 | 2.25E-29 |
| Plant-pathogen interaction | ko04626 | 15 | 0.024451478 |
| Purine metabolism | ko00230 | 12 | 0.007647636 |
| RNA degradation | ko03018 | 10 | 0.011931557 |
| Glutathione metabolism | ko00480 | 10 | 0.000432235 |
| Spliceosome | ko03040 | 10 | 0.531473709 |
| Starch and sucrose metabolism | ko00500 | 8 | 0.29995037 |
| MAPK signaling pathway | ko04010 | 8 | 0.00043236 |
| Pyrimidine metabolism | ko00240 | 6 | 0.169727766 |
| Glycolysis / Gluconeogenesis | ko00010 | 6 | 0.235836294 |
| **Pathways for down-regulated DEGs** | | | |
| Ribosome | ko03010 | 39 | 1.28E-21 |
| Cysteine and methionine metabolism | ko00270 | 10 | 1.71E-07 |
| Phenylpropanoid biosynthesis | ko00940 | 4 | 0.056963625 |
| Mineral absorption | ko04978 | 4 | 8.33E-07 |
| Starch and sucrose metabolism | ko00500 | 4 | 0.199602076 |
| Glycine, serine and threonine metabolism | ko00260 | 4 | 0.003682945 |
| Oxidative phosphorylation | ko00190 | 3 | 0.383767708 |
| RNA transport | ko03013 | 3 | 0.342215759 |
| Plant-pathogen interaction | ko04626 | 3 | 0.560818432 |
| Amino sugar and nucleotide sugar metabolism | ko00520 | 2 | 0.246875701 |
| **CM2 vs. CM4** | | | |
| **Pathways for up-regulated DEGs** | | | |
| Protein processing in endoplasmic reticulum | ko04141 | 37 | 5.38E-13 |
| Flavonoid biosynthesis | ko00941 | 14 | 2.72E-11 |
| Glutathione metabolism | ko00480 | 11 | 0.000100081 |
| RNA transport | ko03013 | 9 | 0.151275041 |
| Starch and sucrose metabolism | ko00500 | 9 | 0.185756248 |
| Spliceosome | ko03040 | 9 | 0.646741066 |
| Ribosome | ko03010 | 8 | 0.942547412 |
| Purine metabolism | ko00230 | 8 | 0.144915234 |
| Lysosome | ko04142 | 8 | 0.002398081 |
| Pentose and glucuronate interconversions | ko00040 | 7 | 0.001843891 |
| **Pathways for down-regulated DEGs** | | | |
| Ribosome | ko03010 | 42 | 3.99E-12 |
| Oxidative phosphorylation | ko00190 | 13 | 0.007328649 |
| Photosynthesis - antenna proteins | ko00196 | 11 | 2.75E-10 |
| Starch and sucrose metabolism | ko00500 | 11 | 0.033160947 |
| Plant-pathogen interaction | ko04626 | 11 | 0.136610131 |
| Protein processing in endoplasmic reticulum | ko04141 | 10 | 0.258637373 |
| Cysteine and methionine metabolism | ko00270 | 10 | 0.000199178 |
| Plant hormone signal transduction | ko04075 | 9 | 0.16436668 |
| Amino sugar and nucleotide sugar metabolism | ko00520 | 7 | 0.03025147 |
| Carbon fixation in photosynthetic organisms | ko00710 | 7 | 0.026129294 |
| **CM3 vs. CM4** | | | |
| **Pathways for up-regulated DEGs** | | | |
| Flavonoid biosynthesis | ko00941 | 16 | 3.52E-25 |
| Phenylpropanoid biosynthesis | ko00940 | 6 | 7.03E-05 |
| Ribosome | ko03010 | 5 | 0.075267289 |
| Porphyrin and chlorophyll metabolism | ko00860 | 4 | 5.75E-05 |
| Circadian rhythm - plant | ko04712 | 4 | 2.30E-05 |
| RNA transport | ko03013 | 4 | 0.014997554 |
| Cysteine and methionine metabolism | ko00270 | 4 | 0.000624749 |
| Mineral absorption | ko04978 | 4 | 2.33E-08 |
| Starch and sucrose metabolism | ko00500 | 4 | 0.018064639 |
| Pentose and glucuronate interconversions | ko00040 | 4 | 0.000111599 |
| **Pathways for down-regulated DEGs** |  |  |  |
| Protein processing in endoplasmic reticulum | ko04141 | 46 | 4.23E-28 |
| Photosynthesis - antenna proteins | ko00196 | 12 | 1.57E-13 |
| MAPK signaling pathway | ko04010 | 10 | 1.76E-07 |
| Spliceosome | ko03040 | 10 | 0.084069071 |
| Plant-pathogen interaction | ko04626 | 9 | 0.067479912 |
| Photosynthesis | ko00195 | 6 | 0.000310291 |
| RNA degradation | ko03018 | 6 | 0.038261452 |
| Oxidative phosphorylation | ko00190 | 4 | 0.48761214 |
| Protein export | ko03060 | 4 | 0.010808328 |
| Carbon fixation in photosynthetic organisms | ko00710 | 4 | 0.099563016 |

Note: CM1, large green fruit; CM2, white fruit; CM3, initial red fruit; CM4, partial red fruit

**
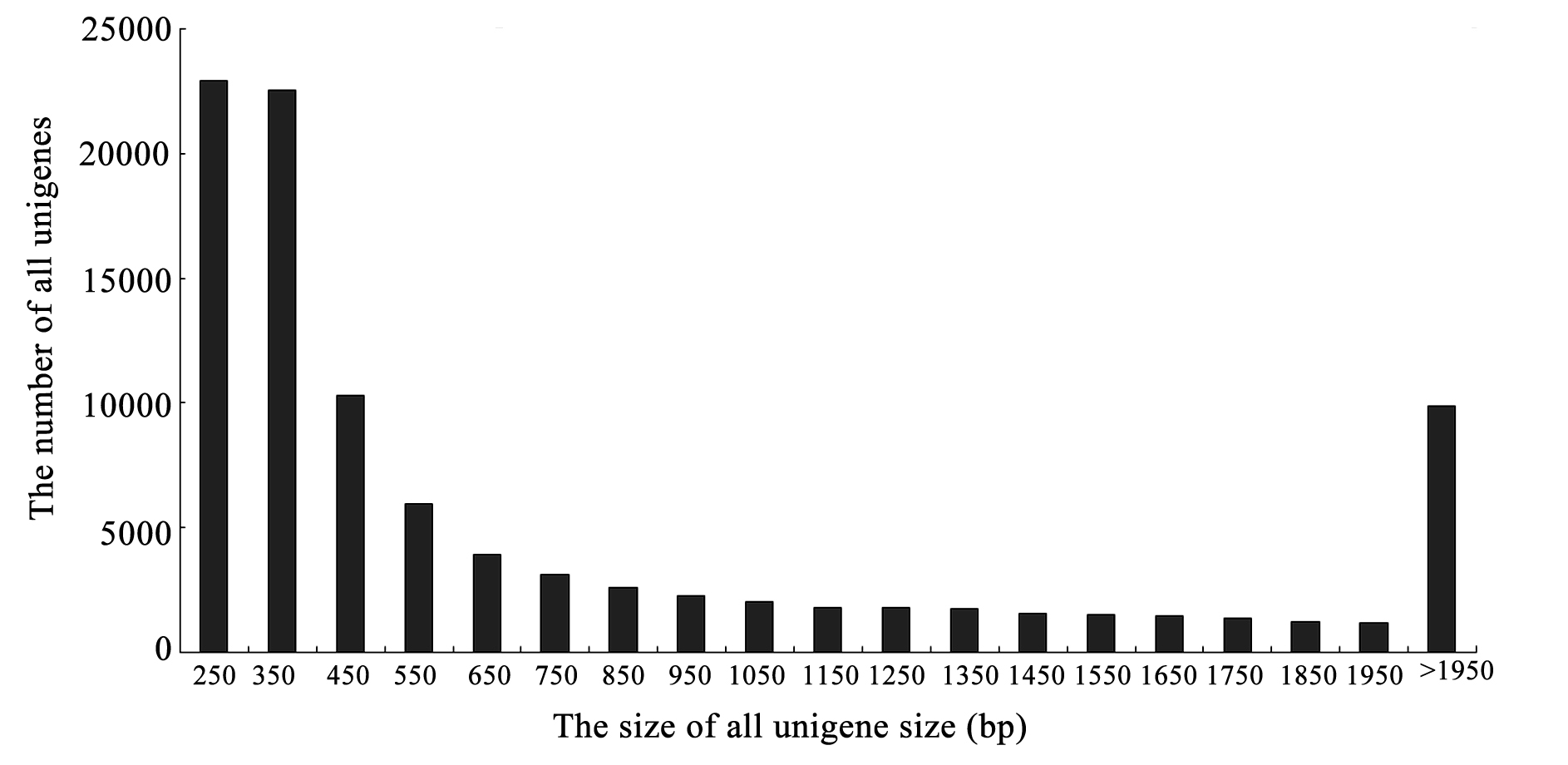
**

**Supplementary Figure 1. Sequence assembly by all-unigene size distribution of strawberry unigenes.**

**
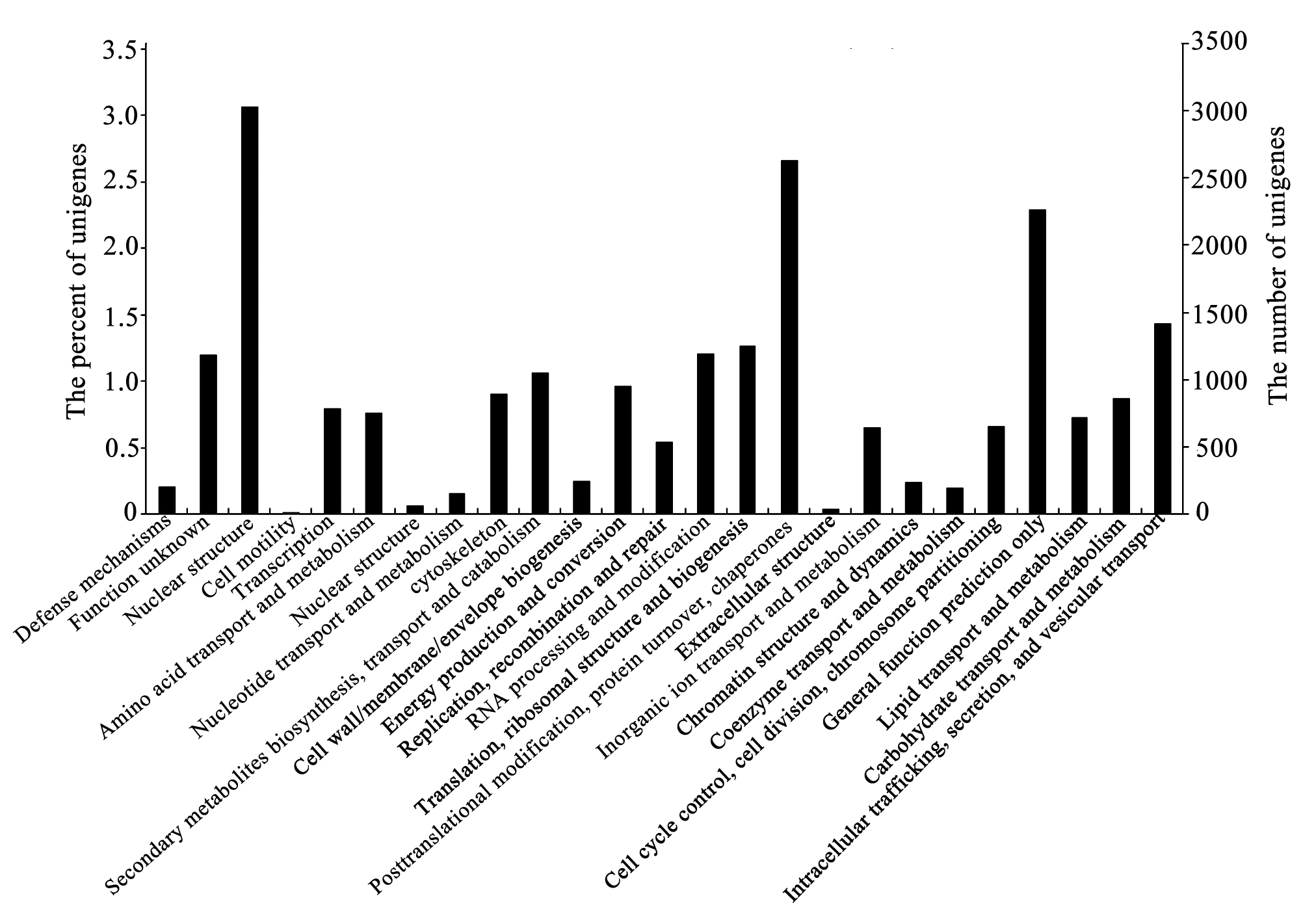
**

**Supplementary Figure 2. KOG function classification of consensus sequence.**

**
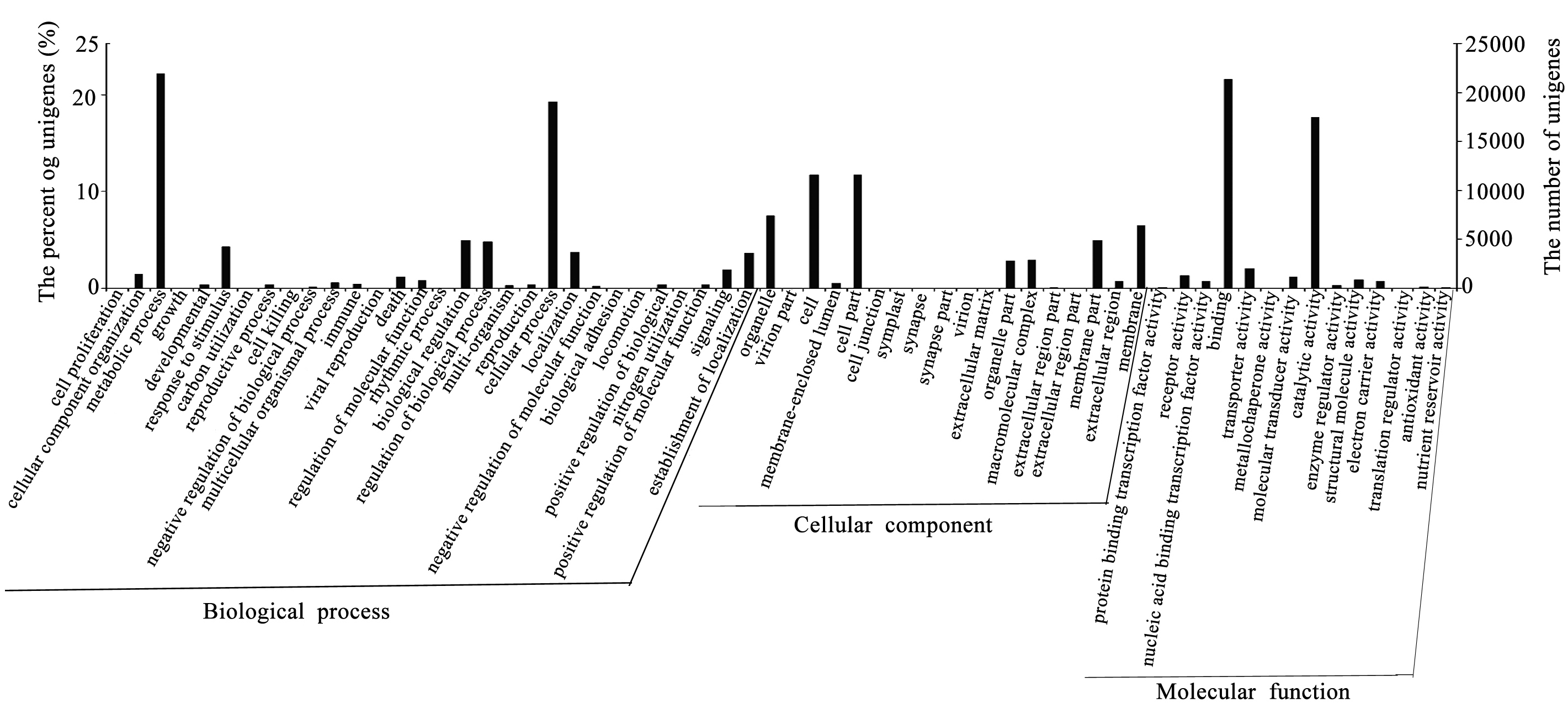
**

**Supplementary Figure 3. The result of comparison of GO classification.** Sample’s function of biological process classification mainly clustered on cellular process and metabolic process; function of cellular component mainly clustered on cell part, and cell and molecular function mainly clustered on binding and catalytic activity.
